# Methods for Efficient Elimination of Mitochondrial DNA from Cultured Cells

**DOI:** 10.1371/journal.pone.0154684

**Published:** 2016-05-02

**Authors:** Domenico Spadafora, Nataliya Kozhukhar, Vladimir N. Chouljenko, Konstantin G. Kousoulas, Mikhail F. Alexeyev

**Affiliations:** 1 Department of Pharmacology, University of South Alabama, Mobile, Alabama, United States of America; 2 Department of Physiology and Cell Biology, University of South Alabama, Mobile, Alabama, United States of America; 3 Division of Biotechnology & Molecular Medicine and Department of Pathobiological Sciences, School of Veterinary Medicine, Louisiana State University, Baton Rouge, Louisiana, United States of America; 4 Center for Lung Biology, University of South Alabama, Mobile, Alabama, United States of America; University of Texas Health Science Center at San Antonio, UNITED STATES

## Abstract

Here, we document that persistent mitochondria DNA (mtDNA) damage due to mitochondrial overexpression of the Y147A mutant uracil-N-glycosylase as well as mitochondrial overexpression of bacterial Exonuclease III or Herpes Simplex Virus protein UL12.5M185 can induce a complete loss of mtDNA (ρ^0^ phenotype) without compromising the viability of cells cultured in media supplemented with uridine and pyruvate. Furthermore, we use these observations to develop rapid, sequence-independent methods for the elimination of mtDNA, and demonstrate utility of these methods for generating ρ^0^ cells of human, mouse and rat origin. We also demonstrate that ρ^0^ cells generated by each of these three methods can serve as recipients of mtDNA in fusions with enucleated cells.

## Introduction

In most mammalian cells, mitochondria generate the bulk of ATP required to sustain a plethora of diverse cellular processes. Besides generating ATP, mitochondria also play important roles in intracellular calcium signalling [1], apoptosis [2], reactive oxygen species (ROS) production [3], biosynthesis of heme and iron-sulphur clusters [4, 5], and other cellular processes. Mitochondria are unique among organelles of mammalian cells in that they house genetic information in the form of mitochondrial DNA (mtDNA). The mitochondrial genome is represented by a covalently closed circular, double-stranded molecule, which is 16,569 bp-long in humans.

mtDNA encodes 37 genes (13 polypeptide components of the oxidative phosphorylation (OXPHOS) system, 2 rRNAs and 22 tRNAs) [6, 7]. Since the discovery that mutations in mtDNA can compromise mitochondrial function and lead to defined human pathology [8–10], there has been an intense and persistent interest in the role of these mutations in human health and disease. Over the years, mtDNA mutations have been implicated in neurodegenerative disorders [11], cancer [12], diabetes [13] and aging [14].

Studies of the cellular effects of mtDNA mutations in humans are confounded by the limited availability of patient material and the diversity of the nuclear background, which can profoundly modulate the expression of a mitochondrial defect [15]. Fortunately, the cybrid technology introduced by King and Attardi [16] greatly facilitates studies of mitochondrial disease. This technology takes advantage of cell lines devoid of mtDNA (ρ^0^ cells) which can be used as recipients of mitochondria in fusions with patient platelets or with cytoplasts derived from fibroblasts by extrusion or chemical inactivation of their nuclei [17–19]. The resulting cytoplasmic hybrids (cybrids) have a uniform genetic background, thus facilitating biochemical analyses.

However, cybrid technology has two limitations: 1) isolation of the ρ^0^ cells requires prolonged (as long as 16 weeks [20]) treatment with ethidium bromide (EtBr) followed by cell cloning and analysis of clones for the presence of mtDNA and 2) such long treatments with EtBr can be mutagenic to nuclear DNA (nDNA). To circumvent these limitations, Kukat et al. generated a fusion between mitochondrially targeted EcoRI restriction endonuclease and Enhanced Green Fluorescent Protein (EGFP). When expressed in recipient cells, this fusion construct enters mitochondria and destroys mitochondrial DNA [21]. While this technique represents a considerable advancement over treatment with EtBr, it has limitations. First, overexpression of a mitochondrially targeted protein can compromise its proper mitochondrial localization and result in mistargeting to the cytosol or nucleus [22]. If this protein is a DNA endonuclease, then its nuclear mistargeting may lead to cytotoxic and mutagenic effects. Second, the method’s utility is limited to elimination of mitochondrial genomes that contain EcoRI sites.

Here, we report that mitochondrial overexpression of three proteins, *Escherichia coli* exonuclease III (ExoIII), mutant Y147A human uracil-N-glycosylase (mUNG1) and Herpes Simplex Virus 1 (HSV-1) protein UL12.5M185, can lead to a complete loss of mtDNA. The latter two proteins efficiently induced the ρ^0^ phenotype in recipient cells when delivered by transient transfection, thus establishing the usefulness of this method for the generation of ρ^0^ cells.

## Materials and Methods

### Cells, viruses and DNA constructs

All cells were propagated in Dulbecco’s Modified Eagle Medium (DMEM) containing 10% Fetal Bovine Serum, 50 μg/ml gentamycin (Invivogen cat# G-1068-50), 50 μg/ml uridine (ThermoFisher Scientific cat# AC140770250), and 1 mM sodium pyruvate (ThermoFisher Scientific cat# MT-25-000-CI) in a humidified atmosphere containing 5% CO_2_ at 37°C, which is permissive for growth of ρ^0^ cells (+UP medium). When indicated, uridine and pyruvate were omitted from this medium for selection of cells containing mtDNA (-UP medium). Doxycycline-inducible lentiviral constructs encoding ExoIII and mUNG1 as well as Tet-On Hela cells transduced with these viruses were described previously [23]. A lentivirus encoding inducible secreted Gaussia luciferase was also described previously [24]. 3T3#52 is a Tet-On derivative of the NIH 3T3 cell line [25]. Plasmids and viral constructs were generated by standard techniques [26] and their diagrams are presented in the S1 Fig. UL12.5M185 protein was cloned by PCR using bacterial artificial chromosome (BAC) vector containing cloned genome of the HSV-1 (McKrae strain [27]) as a template. Plasmids pMA3790 and pMA4008 are available from AddGene (#70110 and #70109, respectively).

### Diagnostics of the ρ^0^ phenotype

The loss of mtDNA was diagnosed by duplex PCR with two pairs of primers (S1 Table): one specific for mtDNA and one specific for nDNA. Due to the high copy number of mtDNA, nDNA is amplified either poorly or not at all in cells retaining mtDNA. The ρ^0^ phenotype was further confirmed in select clones by testing for inability to grow in the absence of uridine and pyruvate. All tested putative ρ^0^ clones were auxotrophic for uridine and pyruvate indicating that methods described herein induce a complete loss of mtDNA rather than introduce mtDNA deletions.

### Production of virus-containing supernatants and transduction of target cells

Lentivirus- and retrovirus-containing supernatants were produced by CaPO_4_-mediated transfection of the HEK293FT and Phoenix Ampho cell lines, respectively, using established protocols [28]. Gag, Pol and Env functions for lentiviral constructs were provided in trans by cotransfecting the vector plasmid with two helper plasmids, psPAX2 and pMD2.G (Addgene cat #12260 and #12259, respectively). Target cells were infected with viruses in 24-well plates or in 35-mm dishes at 30% confluence by incubating them overnight with corresponding supernatant in the presence of 10 μg/mL polybrene (Sigma-Aldrich Corp., St. Louis, MO cat# H9268). The next day, the supernatant was removed and cells were allowed to recover for 24h in DMEM, after which cells were trypsinized and transferred into 150-mm dishes, and antibiotic selection (G418, 1,000 μg/mL; puromycin, 3 μg/mL) was applied for 6 days.

### Luciferase assays

Cells were plated into 24-well plates at 50,000 cells/well, allowed to attach for 2h, and transduced with a lentivirus #2706 [24]. The next day, the medium was changed to one containing or not containing doxycycline at 4 μg/ml. Luciferase was assayed in the supernatants of induced and uninduced cells 24h after induction using BioLux^®^ Gaussia Luciferase Assay Kit (New England Biolabs cat#E3300S) according to manufacturer’s recommendations.

### Transfection

Plasmid transfection was performed using X-fect reagent (Clontech, cat# 631317) according to manufacturer’s recommendations.

### Western blotting

Protein extracts from treated and control cells were prepared using lysis solution containing 10 mM Tris-HCl, 1% SDS, 1x EDTA-free protease inhibitor cocktail (Sigma-Aldrich cat# 11873580001). Protein concentrations were measured using the BCA assay (Thermo Scientific Pierce cat# 23227). Proteins were separated by polyacrylamide gel electrophoresis and transferred to PVDF membranes, blocked and incubated with primary and secondary antibodies using standard techniques [26]. Blots were developed with SuperSignal West Pico and exposed to CL-Xposure film (Thermo Scientific Pierce cat# 34080 and #34088, respectively). Primary antibodies were α-myc tag (Cell Signaling Tehnology cat# 2276), α-HSP60 (mitochondrial, BD Biosciences cat# 611562), and α-cytochrome oxidase subunit 2 (AbCam cat#ab91317).

### Mitochondrial membrane potential

Membrane potential was measured with tetramethylrhodamine methyl ester TMRM (50 nM, ThermoFisher Scientific cat# T668) in the presence or absence of 200 nM valinomycin (ThermoFisher Scientific cat# 33-731-0) or 1 μM CCCP (Sigma-Aldrich cat# C2759) as described previously [23]. It was expressed as uncoupler-sensitive relative fluorescence units (a difference between fluorescence without and with uncoupler).

### Mitochondrial ROS

ROS were measured with MitoSOX Red (ThermoFisher Scientific cat# M36008). Cells were loaded with 5 μM MitoSOX in DMEM for 30 min at 37°C in an atmosphere of 5% (v/v) CO_2_. After treatment, cells were washed, trypsinized and immediately subjected to flow cytometry on a BD FACS Aria with excitation at 561 nm and an emission bandpass filter 582/15. The amount of ROS produced was expressed as percent fluorescence relative to values observed in untreated cells.

### Mitochondrial respiration

Mitochondrial oxygen consumption was measured using Seahorse XF24 extracellular flux analyzer as described previously [29]. Where presented, routine respiration was determined by subtracting non-mitochondrial respiration from baseline respiration.

### Production of transmitochondrial cybrids

Fusions between ρ^0^ NIH3T3#52 and UNG cells were performed using standard chemical enucleation/PEG fusion approach [17].

### Confocal microscopy

For confocal microscopy, cells were plated into 35 mM glass bottom MatTek dishes, allowed to attach overnight and imaged with a Nikon A1R confocal microscope using a 60x water immersion objective.

### Outline of a generic protocol for the production of ρ^0^ cells

1. In 35-mm cell culture dishes, set up transfections of the cell line of interest with either pMA3790 or pMA4008 according to recommendations of the manufacturer of the transfection reagent. The former plasmid is more efficient at inducing the ρ^0^ phenotype, the latter is less likely to induce nDNA alterations due to mistargeting.
2. 72 hours after transfection conduct FACS for EGFP-positive cells using mock-transfected cells to set up sorting gates.
3. Collect ~2,000 positive cells and plate 100, 300 and 900 cells into 150-mm dishes using medium supplemented with uridine (50 ug/ml) and pyruvate (1 mM).
4. Place dishes into a CO_2_ incubator, and observe colony formation.
5. In 2–3 weeks, pick up colonies (e.g., by using cloning disks ThermoFisher Scientific cat# NC9417109) into 24-well plates.
6. When cells reach >20% confluence, trypsinize them with 80 ul of trypsin-EDTA
7. Remove 10 μl of cell suspension for analysis by direct PCR (Viagen cat# 301-C) using duplex PCR with primers specific to mtDNA and nDNA. Add 1 ml of fresh medium supplemented with uridine and pyruvate to remaining cells.
8. Expand putative ρ^0^ cells and confirm the ρ^0^ phenotype by testing them for inability to grow in the absence of uridine and pyruvate.

## Results

### Persistent mtDNA damage can induce the ρ^0^ phenotype in human cells

**Fig 1:**
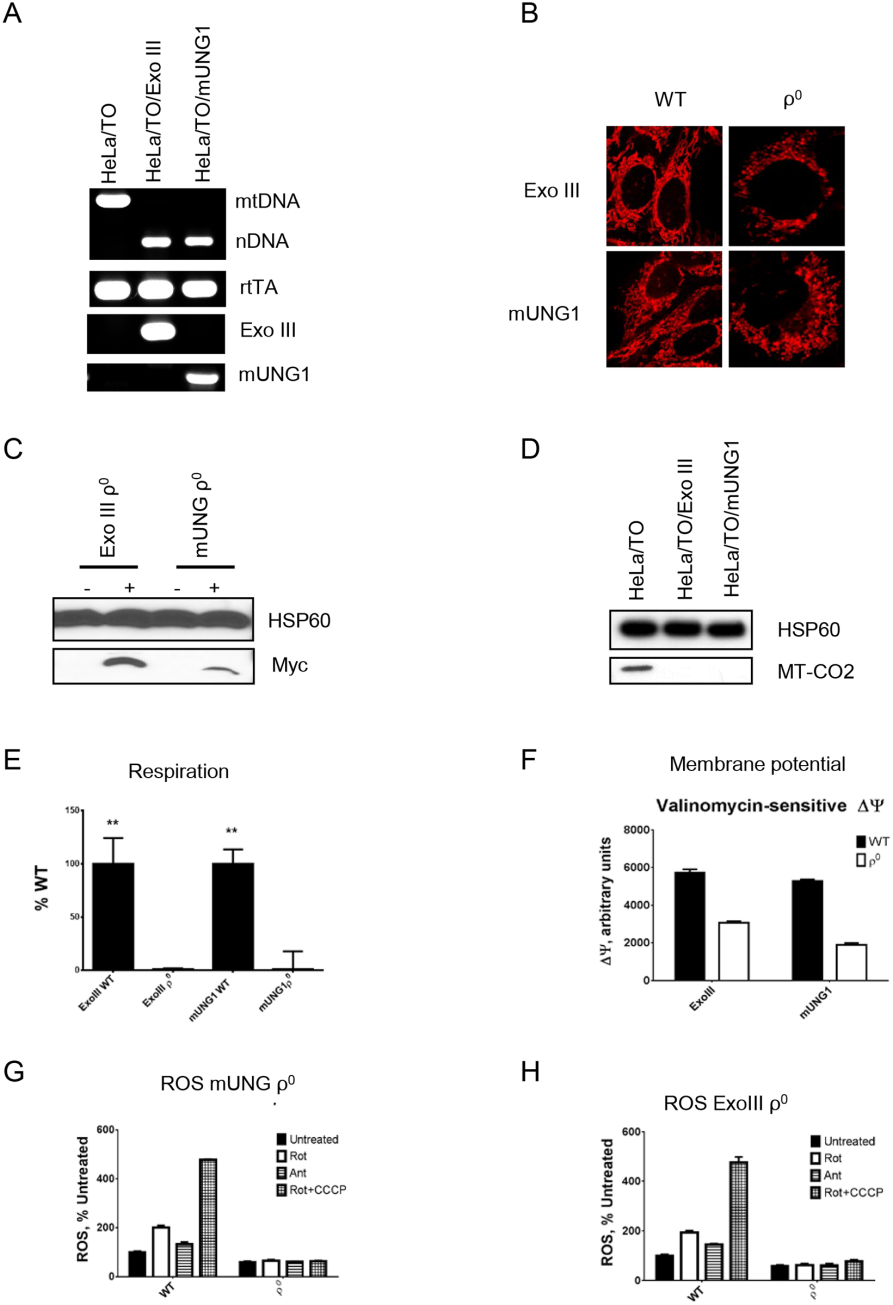
Properties of mtDNA-damage induced ρ^0^ clones. A, ρ^0^ clones have lost their mtDNA, but preserved Tet-On system and transduced ExoIII or mUNG1. B, The mitochondrial network is broken in the ρ^0^ cells, and mitochondria are rounded. C, ρ^0^ clones preserved inducible expression of either ExoIII or mUNG1 (both are myc-tagged). D, Loss of expression of the mtDNA-encoded MT-CO2 subunit in the ρ^0^ cells. E, respiration in the parental and ρ^0^ cells. F, Reduction in the valinomycin-sensitive mitochondrial membrane potential in the mtDNA-damage induced ρ^0^ cells. G and H, reduced mitochondrial ROS production in the ρ^0^ cells. Cells were either left untreated, or treated with rotenone (Rot, 5 μM), antimycin (Ant, 1 μM), or Rot + carbonyl cyanide m-chlorophenyl hydrazine (CCCP, 2 μM). Results are mean ± SEM of at least three biological replicas.

Mitochondrial expression of ExoIII or mUNG1 leads to mtDNA depletion [23]. We explored the long-term consequences of such expression by inducing mitochondrial expression of ExoIII or mUNG1 [23] in HeLa cells for either 12 days or 9 days, respectively. In both instances, cloning of the induced cells resulted in a population of clones heterogenous in size within 18 days of plating. PCR analysis of DNA extracted from those clones after at least 10 days of expansion in 24-well plates revealed that many of the small colonies were formed by cells lacking mtDNA (ρ^0^ cells, Fig 1A).

To better characterise the properties of generated clones, two small clones, generated by induction of ExoIII and mUNG1 respectively, were chosen for further analysis. Regardless of the protein used to induce mtDNA loss, the mitochondrial network in the resulting ρ^0^ cells had a broken morphology, and mitochondria were rounded (Fig 1B). Consistent with retention of both reverse tetracycline-dependent transactivator (rtTA) and either ExoIII or mUNG1 in the resulting clones (Fig 1A), these ρ^0^ cells retained doxycycline-inducible expression of the myc-tagged ExoIII or mUNG1, respectively (Fig 1C). Since mtDNA encodes several subunits of the electron transport chain (ETC), mtDNA loss is always accompanied by a loss of respiration, and by a reduced mitochondrial membrane potential. Indeed, expression of the mtDNA-encoded MT-CO2 subunit was lost in the resulting clones (Fig 1D). Also, a complete loss of respiration was observed in an ExoIII-induced ρ^0^ clone (Fig 1E). The clone with mUNG1-induced mtDNA loss preserved oxygen consumption at ~30% of baseline; however, this rate was unresponsive to inhibitors of the ETC, indicating that this respiration was non-mitochondrial (results not shown). The loss of mtDNA in both cases was accompanied by a reduction in the valinomycin-sensitive mitochondrial membrane potential (Fig 1F).

Reactive oxygen species (ROS) in the form of superoxide anion can be generated as the byproduct of respiration through one-electron reduction of oxygen by ETC [30]. Therefore, reduced mitochondrial ROS production is expected in cells lacking a functional ETC. Indeed, baseline mitochondrial ROS production in the ρ^0^ clones was only 60% of that in parental cells. Furthermore, while ETC inhibitors stimulated mitochondrial ROS production in parental cells, these inhibitors had no effect on mitochondrial ROS production in ρ^0^ cells (Fig 1G and 1H).

**Fig 2:**
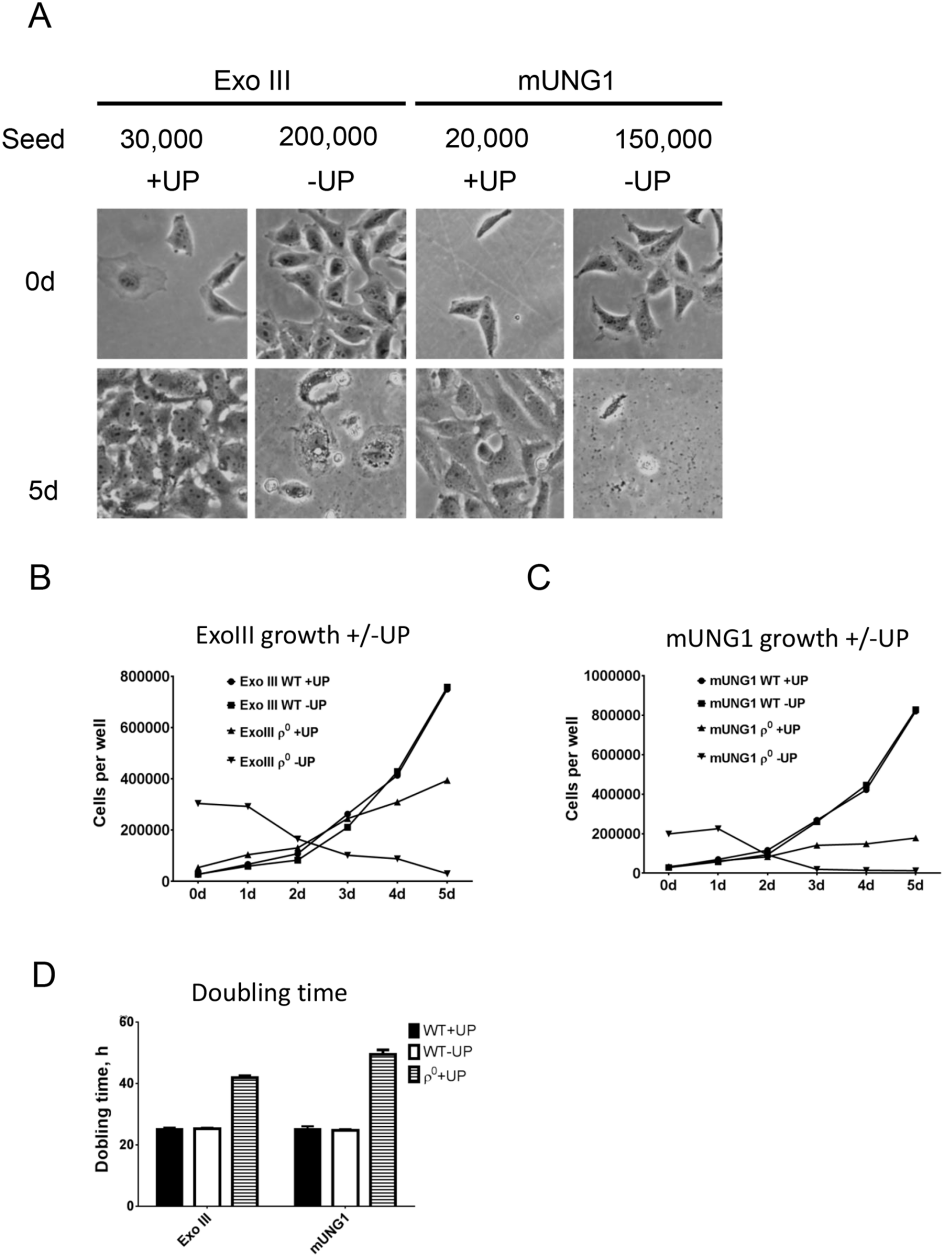
Growth characteristics of the ρ^0^ cells. A, representative field views of the ρ^0^ cells generated with either ExoIII or mUNG1 after seeding (0d) and after growth for 5 days in different media (5d). Seed, number of cells per well at 0d. +/- UP, DMEM medium either supplemented or not with uridine and pyruvate, respectively. B and C, growth curves for the parental (WT) and ρ^0^ cells in different media. D, doubling times for the parental and ρ^0^ cells in different media. Results are mean ± SEM of three biological replicas. Error bars are smaller than symbols.

The loss of mtDNA in cultured cells is typically accompanied by auxotrophy for uridine and pyruvate [16, 31] and by reduced growth rates [32]. Therefore, we examined relative growth rates of obtained ρ^0^ cells in media devoid of uridine and pyruvate and in media supplemented with these compounds. As expected, over the 5-day time course parental cells grew at the same rate regardless of supplementation with uridine and pyruvate. In contrast, ρ^0^ cells grew slowly in media supplemented with uridine and pyruvate and died in unsupplemented media (Fig 2).

### Persistent mtDNA damage can induce ρ^0^ phenotype in mouse cells

**Fig 3:**
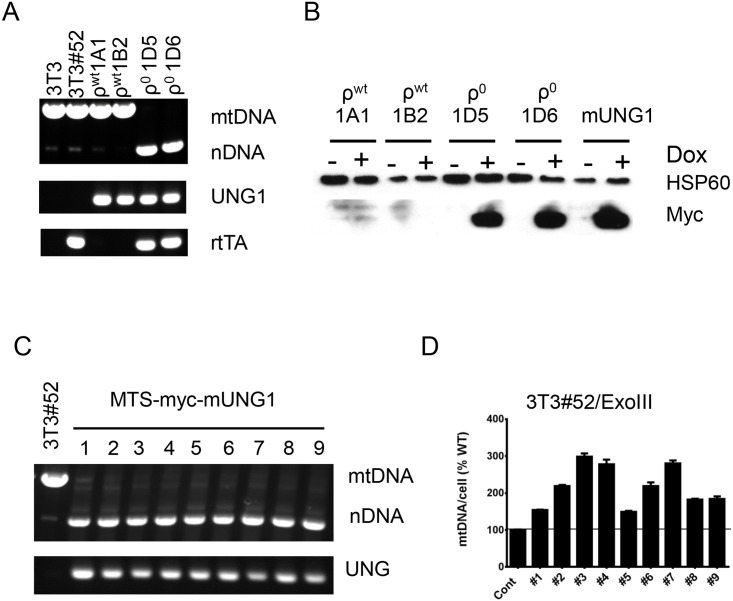
Elimination of mtDNA in mouse cells. A, PCR diagnostics for the presence of the nDNA, mtDNA, UNG1 and rtTA genes in the original NIH3T3, 3T3#52, and in ρ^wt^ and ρ^0^ clones (two each) resulting from transient induction of the mitochondrially targeted mUNG1 in 3T3#52 cells. Note that ρ^wt^ cells lack the rtTA gene. B, detection of the mUNG1 expression in induced and uninduced parental cells (mUNG1) and in the same ρ^wt^ and ρ^0^ clones as in A. Dox, doxycycline; + and—, doxycycline added or not for 24h; HSP60, loading control; myc, myc-taggeg mUNG1. C, PCR analysis of the parental 3T3#52 cells and 3T3#52 cells transduced with a retrovirus encoding mUNG1. D, mtDNA copy number determination in the parental 3T3#52 cells and in these cells transduced with a retrovirus encoding mitochondrially targeted ExoIII. Results are mean ± SEM.

To determine whether persistent mtDNA damage can also induce the ρ^0^ phenotype in mouse cells, we transduced Tet-On 3T3#52 cells [25] with lentivirus encoding inducible mUNG1, induced the resulting cells for 5 days with doxycycline, and identified two types of colonies based on size. The majority of small colonies were formed by ρ^0^ cells, whereas large colonies were formed by cells that retained their mtDNA (ρ^wt^, Fig 3A). To better understand the reasons behind the impairment of the mtDNA elimination process in cells that retained their mtDNA after induction of mUNG1, we analyzed ρ^wt^ and ρ^0^ clones for the presence of mUNG1 and rtTA genes. While both types of clones retained mUNG1 gene, the ρ^wt^ clones had lost their rtTA genes and thus their ability to induce synthesis of mUNG1 in response to treatment with doxycycline (Fig 3A). To confirm this loss of inducibility, ρ^wt^ and ρ^0^ clones were transduced with a lentivirus encoding Tet-On Advanced promoter-controlled secreted Gaussia luciferase, and luciferase activity in supernatants of induced and uninduced cells was measured. Indeed, ρ^wt^ cells had minimal luciferase activity in their supernatants regardless of induction, whereas ρ^0^ clones retained inducibility (S2 Fig). This observation was further confirmed by western blotting of the uninduced and induced ρ^wt^ and ρ^0^ cells, which showed that ρ^wt^ cells failed to induce synthesis of the myc-tagged mUNG1 (Fig 3B).

Therefore, mtDNA elimination was compromised in cells which failed to express mUNG1 due to a loss (presumably, due to genomic instability) of one component of the two-component Tet-On system. Hence, we sought to determine whether a single-vector system will provide a more efficient means for inducing a ρ^0^ phenotype. Towards this end, we generated retroviruses encoding mitochondrially targeted ExoIII and mUNG1 under the control of a weak endogenous long terminal repeat (LTR) promoter (S1 Fig). Of nine randomly chosen clones resulting from transduction of 3T3#52 cells with a mUNG1 retrovirus, all retained the mUNG1 gene and demonstrated a complete loss of mtDNA (Fig 3C). In contrast, all nine analyzed clones resulting from transduction of 3T3#52 cells with a retrovirus encoding mitochondrially targeted ExoIII retained their mtDNA and even demonstrated a moderate increase in mtDNA copy number (Fig 3D). Therefore, Tet-On mediated high-level expression, but not retrovirus-mediated low-level expression, of ExoIII in mitochondria enables establishment of the ρ^0^ phenotype.

### Transient expression of mUNG1 in mitochondria is sufficient for the induction of ρ^0^ phenotype

**Fig 4:**
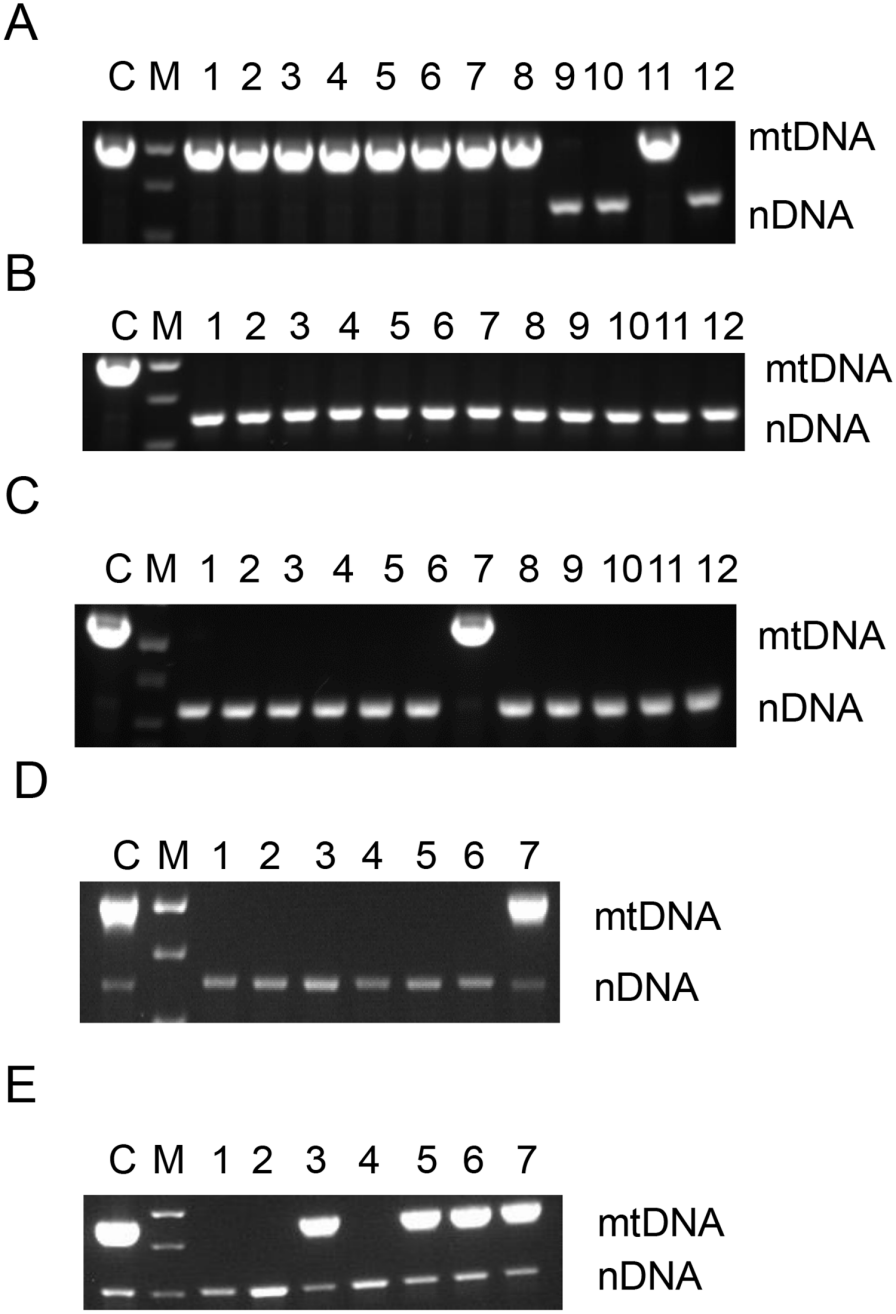
Representative gels of PCR diagnostics of the ρ^0^ phenotype induced by transient transfection. A and B, 3T3#52 cells were transiently transfected with a plasmid encoding both EGFP and mUNG1, and total DNA from the resulting large (A) and small (B) clones was subjected to diagnostic PCR. C, Human osteosarcoma 143B cells were transiently transfected with the same plasmid and analyzed by PCR. D and E, mouse NIH3T3 and rat C6 glioma cells, respectively, were transiently transfected with a plasmid encoding both EGFP and UL12.5M185 protein. M, size marker; mtDNA, a fragment corresponding to mtDNA; nDNA, a fragment corresponding to nDNA. Lane C-in all panels-WT control. Please note that due to the high copy number of mtDNA, nDNA fragment may not amplify in cells containing normal complement of mtDNA.

Transduction with a retrovirus encoding mUNG1 is an efficient means for generating ρ^0^ cells. However, the utility of the resulting cells is limited by their inability to serve as recipients of mtDNA due to persistent expression of mUNG1. Therefore, we sought to test the hypothesis that transient expression of mitochondrially targeted mUNG1 can be used as a means for induction of the ρ^0^ phenotype. In order to test this hypothesis, we constructed a plasmid that expressed both mitochondrially targeted mUNG1 and EGFP (S1 Fig). This plasmid was transiently transfected into 3T3#52 cells, and EGFP-expressing cells were separated by fluorescence-activated cell sorting (FACS) and plated onto 150-mm cell culture dishes. A total of 1053 colonies were identified on 4 plates, of which 12 were large. PCR analysis of total DNA from large colonies revealed that 9 of them retained their mtDNA, whereas all 14 of the small colonies were formed by ρ^0^ cells (Fig 4A and 4B). We repeated this experiment using human osteosarcoma 143B cells and determined that of 25 randomly tested clones, 24 lacked mtDNA (96% efficiency, Fig 4C).

### Transient expression of the HSV UL12.5M185 in mitochondria induces ρ^0^ phenotype

One possible limitation of using Eco RI or mUNG1 for generating ρ^0^ cells is that upon overexpression, a fraction of mitochondrially targeted protein may mislocalize to the cytosol and/or nucleus and thus induce undesirable alterations in the nDNA [33]. In order to overcome this limitation, we explored mitochondrial overexpression of the herpes simplex virus 1 (HSV-1) protein UL12.5, whose truncated form, UL12.5M185, is generated through translation initiation on an alternative methionine residue 185 (M185) and localizes to the mitochondria. In mitochondria, UL12.5M185 may induce mtDNA depletion by a mechanism independent of its nuclease activity [34, 35], that may involve recruitment of the resident mitochondrial nucleases ExoG and EndoG [35]. Therefore, we hypothesized that UL12.5M185 can be used to induce the ρ^0^ phenotype by means of transient transfection. To test this hypothesis, a plasmid encoding both EGFP and UL12.5M185 was constructed (S1 Fig). Upon transient transfection of this plasmid into mouse NIH3T3 and rat C6 glioma cells, the ρ^0^ phenotype was induced with high frequency (85% and 43%, respectively, in representative experiments. Fig 4D and 4E). Unexpectedly, this construct was rather inefficient at inducing ρ^0^ phenotype in human 143B cells. In a representative experiment, 48 colonies were analyzed: 12 large, 12 small, and 24 random. The frequency of ρ^0^ clones was 0% among large colonies, 33% among small colonies, and 9% among randomly picked clones (results not shown).

### ρ^0^ cells generated by all three techniques can serve as recipients of mtDNA

**Fig 5:**
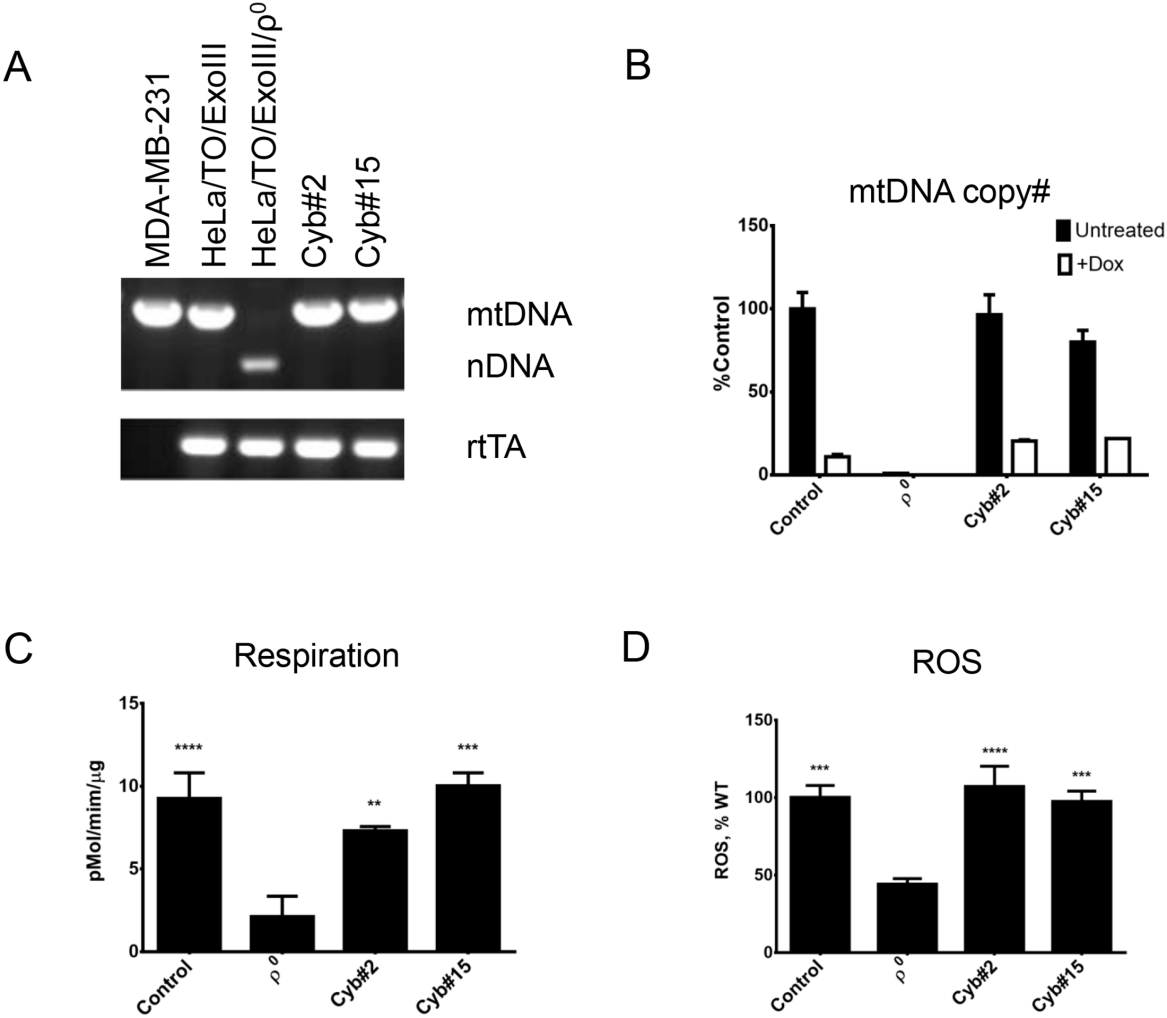
ρ^0^ cells generated by mitochondrial expression of ExoIII can be used for producing cybrids. A, PCR diagnostics of the donor MDA-MB-231 cells, parental HeLa Tet-ON ExoIII cells (HeLa/TO/ExoIII), their derivative obtained by transient induction of mitochondrial ExoIII (HeLa/TO/ExoIII/ρ^0^), and two cybrid clones, #2 and #15. B, cybrids undergo mtDNA depletion upon induction with doxycycline. C and D, Cybrids have elevated respiration and ROS production as compared to recipient ρ^0^ cells. **, P<0.01; ***, P<0.001, ****, P<0.0001 one-way ANOVA.

**Fig 6:**
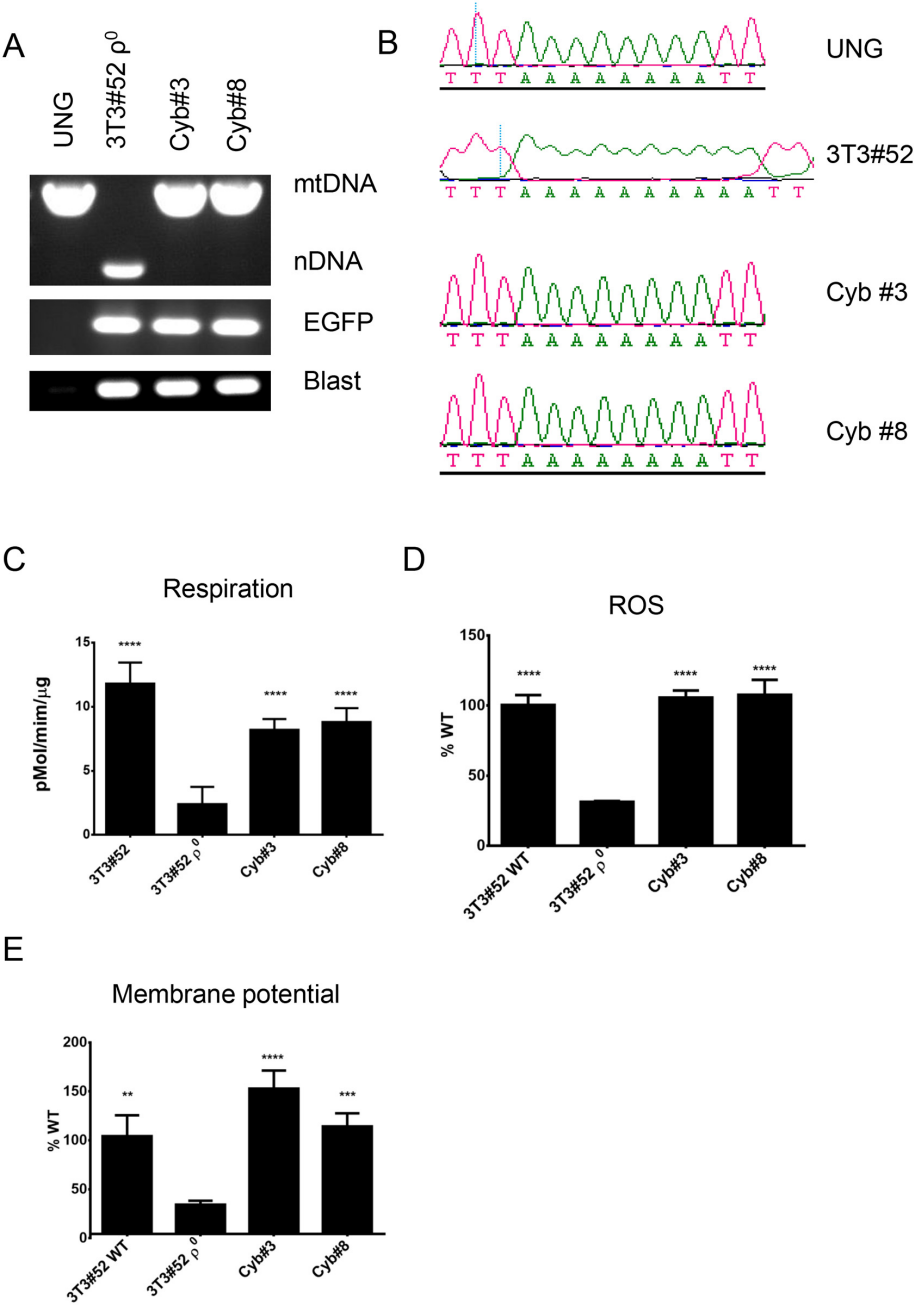
Performance of the ρ^0^ cells generated by mitochondrial expression of mUNG1 in fusions with cytoplasts. A, PCR analysis of cybrids. Cybrids regain mtDNA and retain nuclear markers of recipient 3T3#52 ρ^0^ cells (Blast and EGFP). B, mtDNA sequence in donor, WT recipient and cybrids. Cybrids retain mtDNA polymorphism specific to donor cells. C- E, Respiration, ROS production, and mitochondrial membrane potential in WT recipient, ρ^0^ recipient, and cybrids. **, P<0.01; ***, P<0.001, ****, P<0.0001 one-way ANOVA.

**Fig 7:**
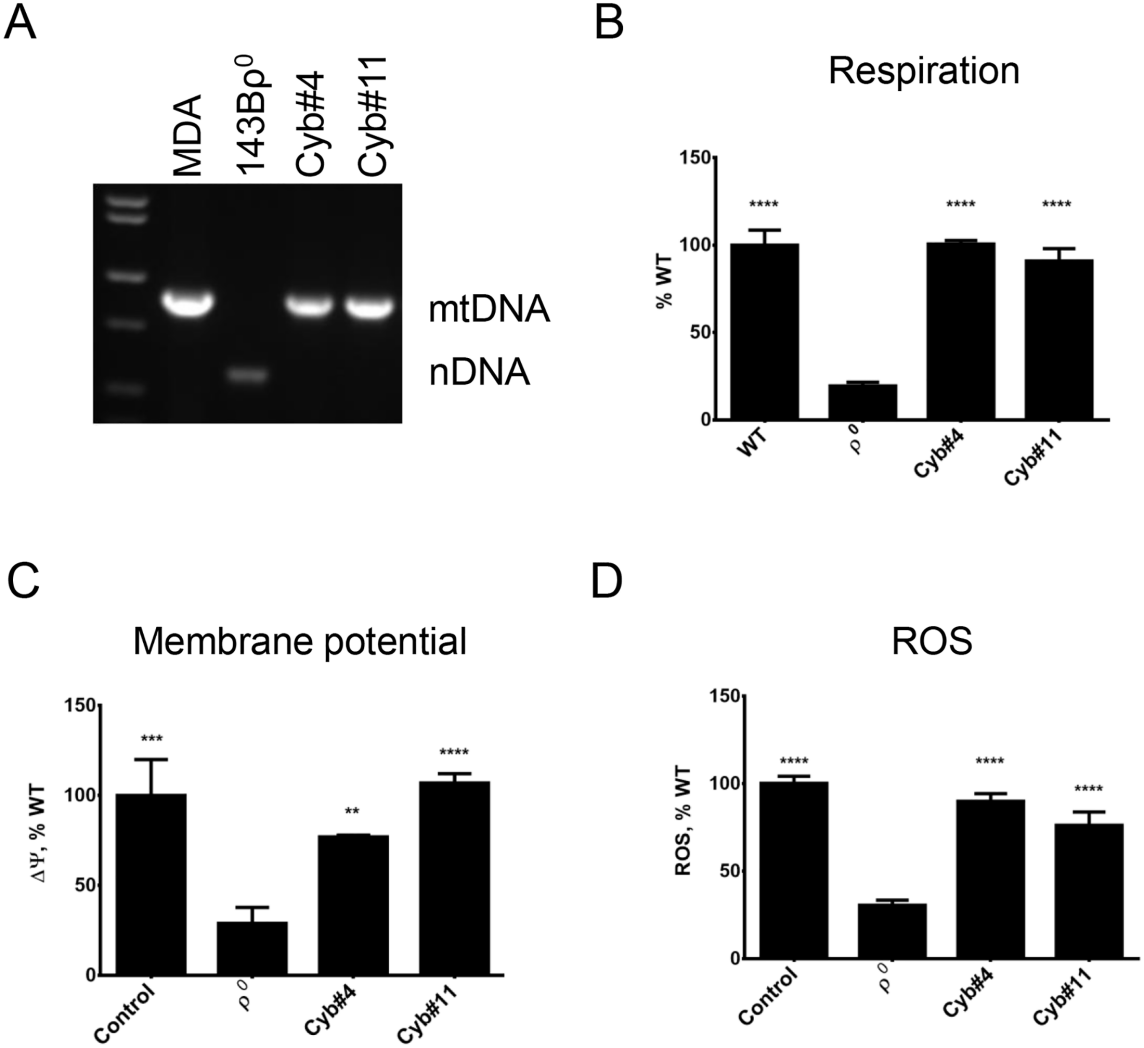
Performance of the ρ^0^ cells generated by mitochondrial expression of UL12.5M185 in fusions with cytoplasts. A, PCR diagnostics of mtDNA content in donor, recipient, and two cybrid clones. B, Respiration of the parental cells and cybrids. C and D, Cybrids have elevated membrane potential and ROS production as compared to recipient ρ^0^ cells. **, P<0.01; ***, P<0.001, ****, P<0.0001 one-way ANOVA.

To evaluate suitability of ρ^0^ cells derived by mitochondrial overexpression of ExoIII, mUNG1 and UL12.5M185 as recipients of mtDNA in cytoplast fusion experiments, we generated cybrids using representative ρ^0^ clones as described in Materials and Methods. HeLa ρ^0^ cells derived by inducible mitochondrial overexpression of ExoIII were fused with chemically enucleated MDA-MB-231 cells. Resulting cybrids retained rtTA, which distinguishes them from donor MDA-MB-231 cells, and obtained mtDNA, which distinguishes them from recipient cells (Fig 5A). Consistent with retention of both rtTA and inducible ExoIII (Fig 1A and 1C), these cells responded to doxycycline induction with reduction of mtDNA copy number (Fig 5B). Consistent with reacquisition of mtDNA and restoration of the ETC function, these cells had significantly increased respiration and ROS production as compared to ρ^0^ cells (Fig 5C and 5D). Similarly, ρ^0^ cells produced by transient transfection of 3T3#52 mouse fibroblasts with pMA3790 encoding mitochondrially targeted mUNG1 were successfully fused with chemically enucleated MEFs to produce cybrids. These cybrids retained nuclear markers of recipient cells (EGFP and blasticidin resistance) and acquired mtDNA, which contains a polymorphism specific to donor (Fig 6A and 6B). As compared to recipient ρ^0^ cells, cybrids had increased respiration, ROS production, and mitochondrial membrane potential. Finally, we induced mtDNA loss in 143B human osteosarcoma cells by transiently transfecting them with pMA4008 (UL12.5M185) and used resulting ρ^0^ cells as recipients in fusions with chemically enucleated MDA-MB-231 cells. As with ρ^0^ cells induced by mitochondrial overexpression of ExoIII and mUNG1, 143B ρ^0^ cells readily produced cybrids, in which reintroduction of mtDNA was accompanied with restoration of respiration and increased mitochondrial membrane potential and ROS production (Fig 7).

## Discussion

Here, we demonstrate that high level of mtDNA damage in the form of abasic sites generated by mitochondrially targeted mUNG1 can be used to induce the ρ^0^ phenotype in cultured cells. Even though mtDNA degradation in response to various types of damage has been demonstrated previously [23, 36–38], this report is the first to demonstrate that mtDNA can be completely lost as a result of persistent damage without concomitant loss of viability. In this report, we harness this novel property of mtDNA in order to develop a method for rapid generation of ρ^0^ cells, which saves weeks compared to traditional mtDNA depletion using EtBr. We also demonstrate that mitochondrial overexpression of the bacterial ExoIII and herpesviral UL12.5M185 can be utilized to achieve the same end in cell lines of human, mouse or rat origin. While the exact mechanism by which ExoIII induces mtDNA depletion remains to be elucidated, it also may involve mtDNA damage through expanding mtDNA nicks into gaps or through antagonizing extension of mtDNA replication primer(s). Overall, these observations provide further support to the notions of mtDNA “dispensability” [39] and its “abandonment” [40].

A 9-day induction of mitochondrially-targeted ExoIII in HeLa cells or a 5-day induction of mUNG1 in 3T3#53 cells resulted in a mixed population of ρ^0^ and ρ^wt^ cells, among which ρ^0^ cells can be identified by their slow growth phenotype (formation of small colonies). However, some cells escaped the loss of mtDNA upon induction of mUNG1. This impairment of mtDNA loss was mediated by a loss of mUNG1 inducibility due to an apparently spontaneous genomic rearrangement resulting in the loss of the rtTA component of the Tet-On system. The ρ^0^ phenotype induced by mitochondrial overexpression of the mUNG1 is identical to that induced by other means, such as prolonged EtBr treatment or EcoRI restriction endonuclease targeting to mitochondria, in that the mitochondrial network is broken, mitochondria are rounded, mitochondrial membrane potential is reduced, cells become auxotrophic for uridine and pyruvate, and expression of mtDNA encoded proteins as well as mitochondrial respiration are lost. Although it was not explicitly demonstrated in this study, we speculate that ρ^0^ cells produced by transient induction of ExoIII and mUNG1 using the Tet-On system can be used for multiple rounds of ρ^0^/cybrid/ρ^0^ cycling due to retention of ExoIII and mUNG1 inducibility. This notion is supported by mtDNA depletion in response to doxycycline induction in cybrids with ExoIII background (Fig 5B).

We also demonstrated that the ρ^0^ phenotype can be efficiently induced by retrovirus-mediated constitutive expression of mUNG1 in mitochondria. Interestingly, induction of the ρ^0^ phenotype by mitochondrial expression of ExoIII was enabled only by high-level expression achieved in the course of Tet-On induction [41], but not by low-level expression mediated by a retroviral LTR promoter, whereas mUNG1 was effective regardless of the level of expression.

We demonstrated that efficient induction of the ρ^0^ phenotype can be achieved by transient expression in mitochondria of either mUNG1 or UL12.5M185 proteins followed by FACS sorting of transfected cells. The efficiency of the method approached 99% in the 3T3#52 cells transiently transfected with mUNG1, and 96% in 143B cells transfected with the same construct. The UL12.5M185 construct produced ρ^0^ cells with somewhat lower efficiency (85% in NIH3T3#52). The ExoIII constructs are the least efficient and appear to induce ρ^0^ phenotype only upon massive mitochondrial overexpression of the protein. However, the exact efficiency is likely to vary between cell lines and transfection conditions, which is suggested by variable efficiency of mUNG1 in different cell lines.

Importantly, the approaches described in this study do not involve extended treatments with potentially mutagenic compounds. Another advantage of these approaches is that they do not depend on the presence of specific restriction endonuclease sites in the mitochondrial genome. Moreover, UL12.5M185-mediated induction of the ρ^0^ phenotype potentially provides a unique advantage of alleviating potential detrimental side effects of nuclear mistargeting. This is because UL12.5M185-mediated mtDNA degradation has been reported to depend on resident mitochondrial nucleases ExoG and EndoG, which are not available in the nucleus [35].

## Conclusions

We described effective methods for the elimination of mtDNA from cultured cells, which are based on mitochondrial overexpression of ExoIII, mUNG1 or UL12.5M185. These methods have several key advantages over currently utilized methodologies and should be useful for rapid generation of ρ^0^ cell lines derived from various tissues of human, rat and mouse origin.

## Supporting Information

S1 Fig Vector maps. A and B, retroviruses encoding mUNG1 and ExoIII, respectively. C, a plasmid encoding EGFP and mUNG1. D, a plasmid encoding UL12.5M185 and EGFP. Abbreviations: amp, bacterial ampicillin resistance gene; BGH pA, HSVTk pA, and SV40 pA, corresponding polyadenylation signals; CMV, EF1a, RSV, and SV40, corresponding promoters; exoIII, Escherichia coli exonuclease III gene; F1 ori, single-stranded origin of replication of the bacteriophage F1; GAG, retroviral GAG protein; LTR, long terminal repeat; MTS, mitochondrial matrix targeting sequence of human ornithine transcarbamylase (1); myc, myc tag epitope; mUNG1, a gene encoding Y147A mutant of the human UNG1; Neo, G418 and kanamycin resistance gene; ori, bacterial origin of replication; UL12.5M185, a gene encoding corresponding HSV-1 protein; WPRE, woodchuck hepatitis virus posttranscriptional regulatory element.
(PPTX)

S2 Fig Luciferase inducibility in the ρ^0^ and ρ^wt^ clones. Clones resulting from transient induction of mUNG1 in mitochondria of the 3T3#52 cells, were transduced with a lentivirus encoding inducible secreted Gaussia luciferase. Luciferase activity in supernatants of induced and uninduced cells was measured. Please note that luciferase activity is not induced in the supernatants of ρ^wt^ cells, whereas ρ^0^ clones retain inducibility. The data are mean ±SEM of three independent experiments.
(PPTX)

S1 Table Oligonucleotides. (DOC)
