# Supplementary material for: Methods for Efficient Elimination of Mitochondrial DNA from Cultured Cells
Source: PLoS One. 2016 May 2;11(5):e0154684. doi: 10.1371/journal.pone.0154684 (PMC4852919; doi:10.1371/journal.pone.0154684)
Supplement: S1 Fig — A and B, retroviruses encoding mUNG1 and ExoIII, respectively. C, a plasmid encoding EGFP and mUNG1. D, a plasmid encoding UL12.5M185 and EGFP. Abbreviations: amp, bacterial ampicillin resistance gene; BGH pA, HSVTk pA, and SV40 pA, corresponding polyadenylation signals; CMV, EF1a, RSV, and SV40, corresponding promoters; exoIII, Escherichia coli exonuclease III gene; F1 ori, single-stranded origin of replication of the bacteriophage F1; GAG, retroviral GAG protein; LTR, long terminal repeat; MTS, mitochondrial matrix targeting sequence of human ornithine transcarbamylase (1); myc, myc tag epitope; mUNG1, a gene encoding Y147A mutant of the human UNG1; Neo, G418 and kanamycin resistance gene; ori, bacterial origin of replication; UL12.5M185, a gene encoding corresponding HSV-1 protein; WPRE, woodchuck hepatitis virus posttranscriptional regulatory element. (PPTX) [file pone.0154684.s001.pptx]

## Slide 1
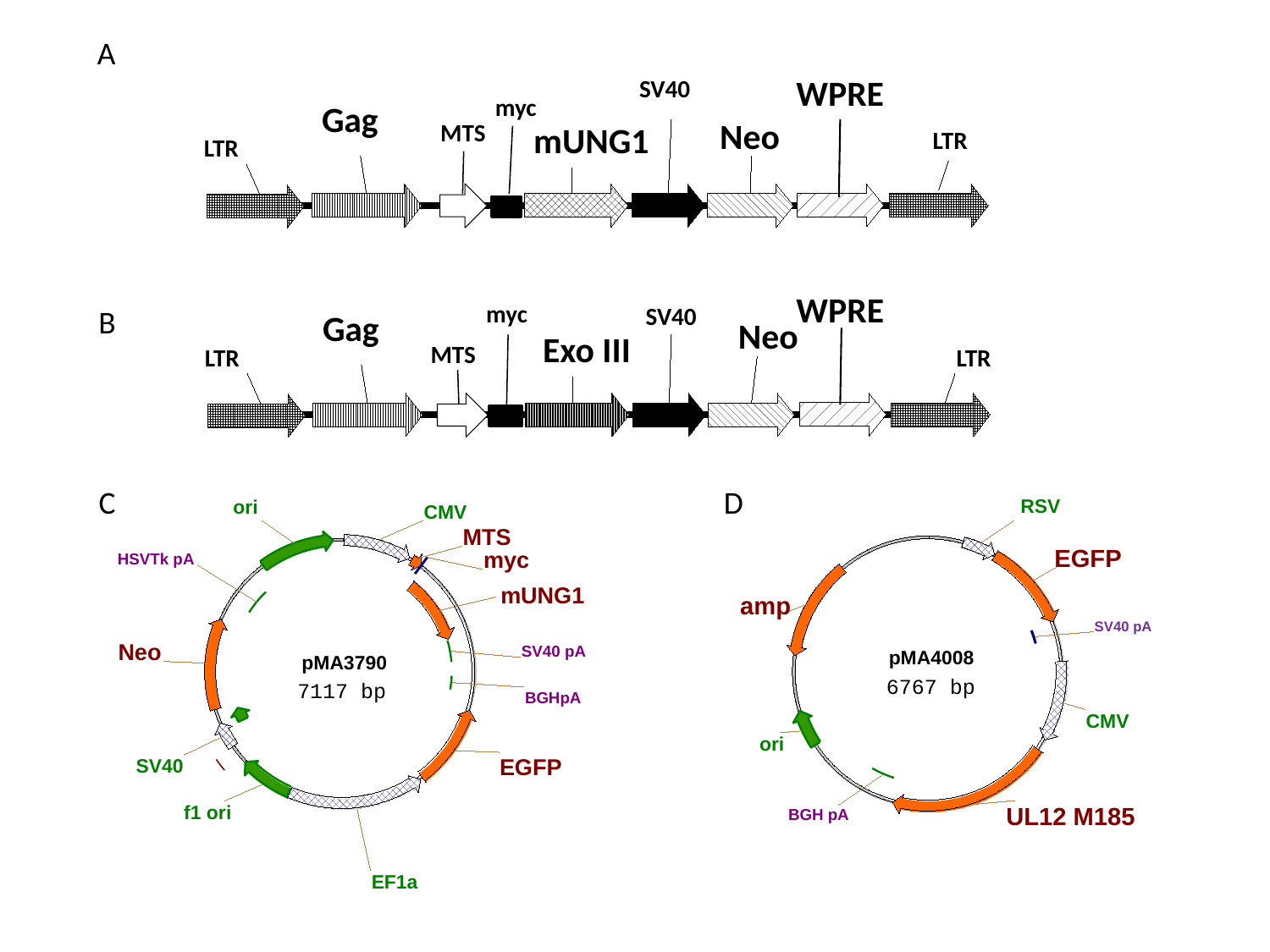

A
WPRE
SV40
myc
Gag
Neo
MTS
mUNG1
LTR
LTR
WPRE
myc
SV40
Gag
Neo
Exo III
MTS
LTR
LTR
B
C
D
RSV
EGFP
amp
SV40 pA
pMA4008
6767 bp
CMV
ori
UL12 M185
BGH pA
ori
CMV
MTS
myc
HSVTk pA
mUNG1
Neo
SV40 pA
pMA3790
7117 bp
BGHpA
EGFP
SV40
f1 ori
EF1a
