# Supplementary figures and images for: Methods for Efficient Elimination of Mitochondrial DNA from Cultured Cells
Source: PLoS One. 2016 May 2;11(5):e0154684. doi: 10.1371/journal.pone.0154684 (PMC4852919; doi:10.1371/journal.pone.0154684)

## Slide 1
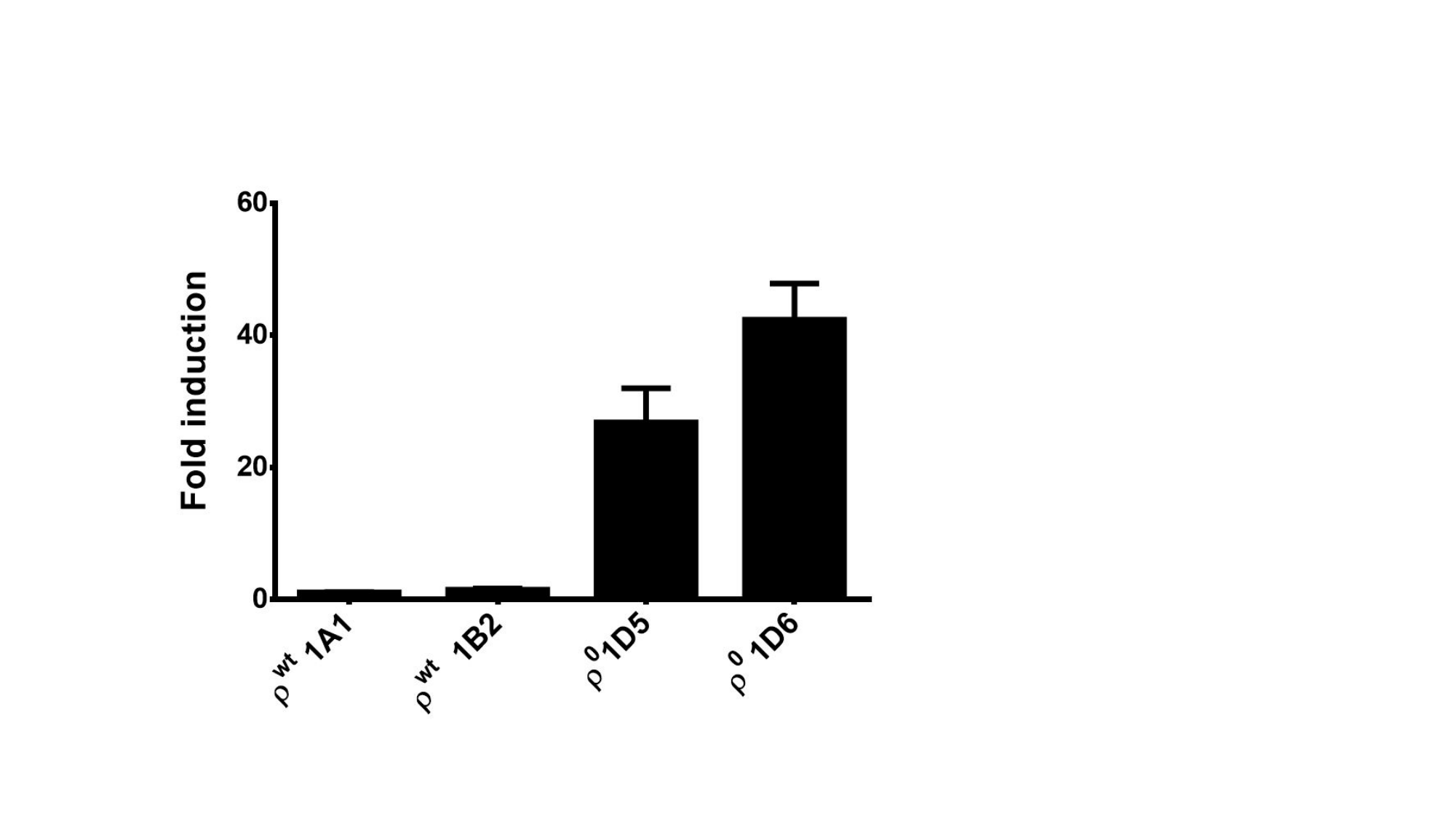

Supplement: S2 Fig — Clones resulting from transient induction of mUNG1 in mitochondria of the 3T3#52 cells, were transduced with a lentivirus encoding inducible secreted Gaussia luciferase. Luciferase activity in supernatants of induced and uninduced cells was measured. Please note that luciferase activity is not induced in the supernatants of ρwt cells, whereas ρ0 clones retain inducibility. The data are mean ±SEM of three independent experiments. (PPTX) [file pone.0154684.s002.pptx]
