# Supplementary material for: Methods for Efficient Elimination of Mitochondrial DNA from Cultured Cells
Source: PLoS One. 2016 May 2;11(5):e0154684. doi: 10.1371/journal.pone.0154684 (PMC4852919; doi:10.1371/journal.pone.0154684)
Supplement: S1 Table — (DOC) [file pone.0154684.s003.doc]

**S1 Table. Oligonucleotides**

| **Purpose** | **Name** | **Sequence** | **Fragment, bp** |
| --- | --- | --- | --- |
| mUNG1 diagnostics in mouse cells | UNGf UNGr | CTTTGGAGAGAGCTGGAAGAAG CTTGGCCCACCCAGATAAAT | 340 |
| mUNG1 diagnostics in HeLa cells | UNG5f UNG5r | CTTTGGAGAGAGCTGGAAGAAG CCTCGGCCTCTGCATAAATAA | 997bp |
| ExoIII diagnostics | ExoIIIf ExoIIIr | CTACCTGGAAACCGAACTCAAA CAGACCACGGTTATCGTCAAA | 277 |
| rtTA diagnostics | rtTAf rtTAr | CGACAAGGAAACTCGCTCAA GATGCTCCTGTTCCTCCAATAC | 378 |
| Diagnostics of ρ^0^ phenotype in mouse cells | mMitF mMitR mNucF mNucR | AAAGCATCTGGCCTACACCCAGAA ACCCTCGTTTAGCCGTTCATGCTA CCACGTGCTCTGTATGAGATT ATGCTGGCTTATCTGTTCCTT | 1041  636 |
| Diagnostics of ρ^0^ phenotype in human cells | HVRF HVRR hNucF hNucR | AATGTCTGCACAGCCACTTTCCAC TCGTAGTGTTCTGGCGAGCAGTTT GTATCTGGGCCTTTGACCTTAC GGTCCACTCCTGACTCTTATCT | 901  467 |
| Diagnostics of ρ^0^ phenotype in rat cells | rMitF rMitR rNucF rNucR | GAGGCTGGAATCTCCCAATAAG GCCTAGGTTGAGGTTGATAAGG CTTGAGGGCTCTGGAAGTAAAG TGGAGGTCAGAAGAGAGTGTAA | 916  511 |
| Diagnostics of EGFP in 3T3#52 | EGFPf EGFPr | ACGTAAACGGCCACAAGT GGGTGTTCTGCTGGTAGTG | 498 |
| Diagnostics of Blasticidin resistance gene in 3T3#52 | BlastF BlastR | GAAAGAGCAACGGCTACAATC TTAGCCCTCCCACACATAAC | 360 |
